# Improving peer review of systematic reviews by involving librarians and information specialists: protocol for a randomized controlled trial

**DOI:** 10.1186/s13063-021-05738-z

**Published:** 2021-11-11

**Authors:** Melissa L. Rethlefsen, Sara Schroter, Lex M. Bouter, David Moher, Ana Patricia Ayala, Jamie J. Kirkham, Maurice P. Zeegers

**Affiliations:** 1 Health Sciences Library & Informatics Center, University of New Mexico, MSC 09 5100, 1 University of New Mexico, Albuquerque, NM 87131-0001 USA; 2 BMJ Publishing Group, London, England; 3 Department of Philosophy, Faculty of Humanities, Vrije Universiteit Amsterdam, De Boelelaan 1105, 1081 HV Amsterdam, The Netherlands; 4 Department of Epidemiology and Data Science, Amsterdam UMC, Vrije Universiteit Amsterdam, De Boelelaan 1089a, 1081 HV Amsterdam, The Netherlands; 5 Centre for Journalology, Clinical Epidemiology Program, Ottawa Hospital Research Institute, The Ottawa Hospital, General Campus, Centre for Practice Changing Research Building, 501 Smyth Road, PO BOX 201B, Ottawa, Ontario K1H 8L6 Canada; 6 Gerstein Science Information Centre, University of Toronto, Toronto, Canada; 7 Centre for Biostatistics, Manchester Academic Health Science Centre, University of Manchester, Manchester, UK; 8 Department of Epidemiology, School for Nutrition and Translational Research in Metabolisms, Care and Health Research Institute, Maastricht University Medical Center+, PO Box 616, 6200 MD Maastricht, The Netherlands

**Keywords:** Peer review, Librarians and information specialists, Systematic reviews, Literature searching

## Abstract

**Background:**

Problems continue to exist with the reporting quality and risk of bias in search methods and strategies in systematic reviews and related review types. Peer reviewers who are not familiar with what is required to transparently and fully report a search may not be prepared to review the search components of systematic reviews, nor may they know what is likely to introduce bias into a search. Librarians and information specialists, who have expertise in searching, may offer specialized knowledge that would help improve systematic review search reporting and lessen risk of bias, but they are underutilized as methodological peer reviewers.

**Methods:**

This study will evaluate the effect of adding librarians and information specialists as methodological peer reviewers on the quality of search reporting and risk of bias in systematic review searches. The study will be a pragmatic randomized controlled trial using 150 systematic review manuscripts submitted to *BMJ* and *BMJ Open* as the unit of randomization. Manuscripts that report on completed systematic reviews and related review types and have been sent for peer review are eligible. For each manuscript randomized to the intervention, a librarian/information specialist will be invited as an additional peer reviewer using standard practices for each journal. First revision manuscripts will be assessed in duplicate for reporting quality and risk of bias, using adherence to 4 items from PRISMA-S and assessors’ judgements on 4 signaling questions from ROBIS Domain 2, respectively. Identifying information from the manuscripts will be removed prior to assessment.

**Discussion:**

The primary outcomes for this study are quality of reporting as indicated by differences in the proportion of adequately reported searches in first revision manuscripts between intervention and control groups and risk of bias as indicated by differences in the proportions of first revision manuscripts with high, low, and unclear bias. If the intervention demonstrates an effect on search reporting or bias, this may indicate a need for journal editors to work with librarians and information specialists as methodological peer reviewers.

**Trial registration:**

Open Science Framework. Registered on June 17, 2021, at 10.17605/OSF.IO/W4CK2.

## Administrative information

Note: the numbers in curly brackets in this protocol refer to SPIRIT checklist item numbers. The order of the items has been modified to group similar items (see http://www.equator-network.org/reporting-guidelines/spirit-2013-statement-defining-standard-protocol-items-for-clinical-trials/).

**Table:** 

Title {1}	Improving peer review of systematic reviews by involving librarians and information specialists: protocol for a randomized controlled trial
Trial registration {2a and 2b}.	This trial was registered on the Open Science Framework on June 17, 2021 at 10.17605/OSF.IO/W4CK2.
Protocol version {3}	1
Funding {4}	No specific funding was received for this study. It is part of MLR’s self-funded PhD project registered at Maastricht University, the Netherlands, in collaboration with the British Medical Journal, United Kingdom.
Author details {5a}	Melissa L. Rethlefsen Health Sciences Library & Informatics Center, University of New Mexico MSC 09 5100, 1 University of New Mexico, Albuquerque, NM 87131-0001 mlrethlefsen@gmail.com https://orcid.org/0000-0001-5322-9368 Sara Schroter BMJ Publishing Group London, England https://orcid.org/0000-0002-8791-8564 Lex M. Bouter Department of Philosophy, Faculty of Humanities, Vrije Universiteit Amsterdam, De Boelelaan 1105, 1081 HV, Amsterdam, The Netherlands Department of Epidemiology and Data Science, Amsterdam UMC, Vrije Universiteit Amsterdam, De Boelelaan 1089a, 1081 HV, Amsterdam, The Netherlands lm.bouter@vu.nl https://orcid.org/0000-0002-2659-5482 David Moher Centre for Journalology, Clinical Epidemiology Program, Ottawa Hospital Research Institute, The Ottawa Hospital, General Campus, Centre for Practice Changing Research Building, 501 Smyth Road, PO BOX 201B, Ottawa, Ontario, K1H 8L6, Canada https://orcid.org/0000-0003-2434-4206 Ana Patricia Ayala Gerstein Science Information Centre, University of Toronto, Toronto, Canada https://orcid.org/0000-0002-3613-2270 Jamie J. Kirkham Centre for Biostatistics, Manchester Academic Health Science Centre, University of Manchester, Manchester, UK https://orcid.org/0000-0003-2579-9325 Maurice P. Zeegers Department of Epidemiology, School for Nutrition and Translational Research in Metabolisms, Care and Health Research Institute, Maastricht University Medical Center+, PO Box 616, 6200, MD, Maastricht, The Netherlands https://orcid.org/0000-0002-2387-083X
Name and contact information for the trial sponsor {5b}	Not applicable, no trial sponsor
Role of sponsor {5c}	Not applicable, no trial sponsor

## Introduction

### Background and rationale {6a}

Systematic reviews exist to collect and synthesize the evidence on a research question, using formal and explicit methods and eligibility criteria that serve to limit the bias in and to improve the replicability of the review. This evidence is collected through a search process that seeks to identify all studies that meet the eligibility criteria [1, 2]. Identifying all eligible studies requires systematic review teams to use highly sensitive searches in diverse databases and information sources, such as trial registries [3]. Because of the complexity of the search process, systematic review guidelines recommend the inclusion of an information specialist or librarian on the systematic review team to design and conduct the searches [2, 4]. Guidelines for reporting systematic review protocols and completed reviews also include detailed guidance on how to report searches to ensure reproducibility and to allow readers to assess bias that may have been introduced into the search through choices made in information sources and search strategies [3, 5–8].

Though guidelines to promote both information sources and techniques designed to retrieve as many eligible studies as possible exist, there is substantial evidence that many systematic reviews are at high risk of bias due to the limited number of information sources selected, the lack of sensitive searching, or both [9–11]. In addition, though reporting guidelines have explicitly described what components of a search are necessary to describe fully, inadequate reporting continues to be a major problem [12–17]. The Preferred Reporting Items for Systematic reviews and Meta-Analysis Statement (PRISMA Statement), which was originally published in 2009, has been cited over 36,000 times [18], for example, yet over 60% papers that claim to adhere to its guidelines for reporting fail to present a complete search strategy which is called for by Item 8 in the PRISMA Statement checklist [9].

One of the reasons that systematic reviews with poorly reported searches or searches that are at high risk of bias are published may be that peer reviewers do not have the methodological expertise to review the search components of systematic reviews. Another may be that peer reviewers are commenting upon searches when reviewing, but that the comments are not being fully addressed by authors. Additionally, peer reviewers may limit their comments to certain areas, such as date ranges, that may be too superficial or too limited to address aspects of the search that profoundly impact quality, such as breadth of information sources or the sensitivity of search strategies [19, 20]. There is also potential that peer reviewers perceive the peer review process as being too late for systematic review teams to address concerns about the risk of bias introduced by the search or a belief that regardless of the perceived risk of bias, that the search could have identified all relevant citations. Peer reviewers may not test or rerun searches themselves, relying on a cursory examination of the search [19, 20].

Though the reason is not fully understood, it also is clear that the poor quality of reporting of searches means that many simple errors appear in published systematic reviews. Though the original search may have identified all relevant articles, poor reporting directly impacts reproducibility. Clarity of reporting may also reduce the perceived risk of bias. For example, authors may state they searched a platform, but do not mention any of the databases searched, or authors may not distinguish between keywords and Medical Subject Headings (MeSH terms) [21–23]. For one, it would not be possible to reproduce such searches, and, secondly, it may appear as though the search was poorly done. These errors could be easily identified by librarians and information specialists. Though recommendations from the National Academy of Medicine and the Cochrane Collaboration endorse having librarians and information specialists peer review search strategies prior to conducting the searches to identify these errors, this form of early-stage peer review has not been widely adopted [2, 4, 24]. While ideally errors, omissions, and bias would be identified early [25, 26], peer review at manuscript submission remains an important safeguard, especially since so few systematic reviews have peer-reviewed protocols [9].

Methodological expertise is often recognized by journal editors as a necessity for reviewing complex aspects of research, such as statistical analyses [27–30]. In fact, some journals employ statisticians specifically to perform this form of methodological peer review. Methodological or specialist reviewers, including statistical reviewers, provide comments that are distinct from those made by other reviewers [27]. Only a few studies have assessed the effect of including specialist reviewers in the peer review process [31–34]. One randomized control trial that assessed the impact of statistician peer reviewers found that they improved the quality of reporting biomedical articles [31]. This effect was found to be larger than sending conventional peer reviewers copies of relevant reporting guidelines to consider for their review. A further study conducted in the same journal demonstrated that adding an additional statistical reviewer to specifically address adherence to the appropriate reporting guideline for each study improved the overall quality of the final manuscript [34]. A follow-up study also showed preliminary findings that suggest methodological review may have a positive effect on future citations [32]. More recently, Blanco et al. performed a randomized controlled trial which demonstrated that specialized editorial intervention by a reporting guideline expert improved reporting quality of clinical trials [33].

Librarians and information specialists (LIS), who are experts in search methods, are an underutilized resource for methodological peer review. Like statisticians, LIS have highly specialized expertise that would enable them to provide a detailed methodological review of the search strategy. A thorough methodological review of the search strategy could enable authors to correct errors, improve reporting quality and reproducibility, and reduce the risk of bias in their systematic review by searching additional, recommended information sources or adding a wider range of search terms to increase sensitivity. LIS reviewers may also be able to identify searches with fatal flaws and reject poor-quality manuscripts that cannot be improved. A recent survey of 291 health sciences librarians found that while 22% had been invited to peer review at least one systematic review, an additional 74% would be interested or would consider peer reviewing systematic reviews for journals [35]. This is further evidenced by the presence of the Librarian Peer Reviewer Database [36], a tool created to connect librarians and information specialists who have an interest in peer reviewing systematic reviews and evidence syntheses with journal editors. As of August 2021, this database contained the names and specialties of nearly 150 LIS internationally, largely from the health sciences.

Though LIS have expressed interest in being methodological peer reviewers, there have been no studies to test whether inviting LIS to participate as methodological peer reviewers improves the quality of reporting or reduces the risk of bias in published systematic reviews.

### Objectives {7}

The objective of the study is to evaluate the effect of adding librarians and information specialists as methodological peer reviewers on the quality of search reporting and risk of bias in systematic review searches.

### Trial design {8}

The study will be a pragmatic randomized controlled trial using submitted systematic review manuscripts as the unit of randomization. Submitted manuscripts of completed systematic reviews were selected as the unit of study to reflect the current rarity of both pre-search peer review and protocol registration or publication.

The protocol is reported using the SPIRIT guidelines [37] as a framework.

## Methods: participants, interventions, and outcomes

### Study setting {9}

The study will take place in collaboration with the BMJ Publishing Group.

### Eligibility criteria {10}

Journals published by the BMJ Publishing Group are eligible for the study. We purposively selected two general medical journals (*BMJ* and *BMJ Open*) from BMJ Publishing Group that each published at least 20 systematic reviews in 2020.

#### Manuscript eligibility

All new manuscript submissions describing a systematic review submitted to participating journals (*BMJ* and *BMJ Open*), which the journal editor has decided to send out for peer review will be eligible for inclusion. Manuscripts will be accessed through these journals’ editorial systems, ScholarOne.

To be considered a systematic review manuscript, the manuscript must:

1. Use (or claim to use) a systematic review or related evidence synthesis methodology as its primary methodology. This includes scoping reviews, rapid reviews, and other evidence syntheses that use formal, explicit, and pre-specified methods to conduct the review.

Manuscripts will not be considered if:

1. Systematic review or related evidence synthesis method is not the primary methodology used (for example, if a systematic review is conducted as part of a case report).
2. The manuscript is a meta-analysis that was conducted without a search component, such as the results from a prospective meta-analysis or a systematic review relying solely on clinical study reports.
3. The manuscript is a study protocol.
4. The editor chooses not to send the manuscript for peer review.
5. The manuscript is a republication or an abridged version of a systematic review published elsewhere (i.e., an abridged Cochrane review).
6. It is a fast-track manuscript requiring immediate peer reviewer response.

### Who will take informed consent? {26a}

Not applicable. This study was determined to not be human subjects research by the Institutional Review Board at the University of New Mexico Health Sciences Center.

### Additional consent provisions for collection and use of participant data and biological specimens {26b}

The BMJ Publishing Group’s Company Privacy Statement states that reviews and manuscripts may be used for quality improvement purposes. All authors of submitted manuscripts receive the following notice, customized to the journal: “We are constantly trying to find ways of improving the peer review system and continually monitor processes and methods by including article submissions and reviews in our research. If you do not wish your paper to be entered into our peer review research programme, please let us know by emailing [journal-specific email address] as soon as possible.” In addition, all peer reviewers invited to review by BMJ Publishing Group’s journals receive the following statement as part of their email invitation, customized to the journal: “We are constantly trying to find ways of improving the peer review system and have an ongoing programme of research. If you do not wish your review entered into a study please let us know by emailing [journal-specific email address] as soon as possible.”

## Interventions

### Explanation for the choice of comparators {6b}

The comparator is usual practice at each of the participating journals. This was chosen to understand if adding a methodological peer reviewer to usual practice would result in differences in search reporting quality and risk of bias.

### Intervention description {11a}

Control group (usual practice): Editors will invite peer reviewers using standard practices. Peer reviewers will receive automated, journal-specific standard emails per the journal’s usual practice.

Intervention group: Editors will invite peer reviewers using standard practices. For each manuscript, an LIS reviewer will also be invited using standard practices. The LIS reviewer will receive an identical invitation and instructions when invited to peer review as the other invited peer reviews.

Prior to the study’s commencement, LIS reviewers will be identified by MLR using the Librarian Peer Reviewer Database [36] and added to each journal’s database of available peer reviewers. Any LIS reviewer in the Librarian Peer Reviewer Database who listed an interest in reviewing manuscripts in health and biomedical disciplines will be considered eligible, though they may have differing levels of expertise and experience. LIS reviewer accounts will be color flagged in the ScholarOne system so they can be easily located. Editors will be asked not to use these reviewers for reviewing manuscripts outside of this research study to avoid oversampling them. Study researchers (MLR) and assessors (APA, others to be determined) will be excluded from participation as reviewers during the course of the study.

For each manuscript randomized to the intervention, MLR will flag the manuscript (using a specific colored manuscript account flag visible to editors). MLR will have full access to ScholarOne and, for intervention manuscripts, will manually increase the quota for the number of reviewers required for the manuscript and then invite a flagged LIS reviewer to review alongside the peer reviewers. To avoid oversampling of individual LIS reviewers, MLR will track invitations to LIS reviewers and will not invite a LIS reviewer a second time until all LIS reviewers have been invited to participate in a review. The invitation for LIS reviewers will be the same standard email sent to peer reviewers. LIS reviewers will receive no special instructions.

If the invited LIS reviewer declines the invitation to review a particular manuscript, an additional LIS reviewer will be invited until one LIS reviewer has accepted the review invitation for that manuscript. All reviewers, including the LIS reviewers, will receive automated, journal-specific standard emails per the journal’s usual practice.

After peer review, manuscripts are either rejected or authors are asked to revise their manuscripts for further editorial scrutiny. All randomized manuscripts will be tracked to determine the editorial decision post review (reject or revise). The first revised version after initial peer review will be the version that is assessed for study outcomes relating to the quality of reporting and risk of bias.

### Criteria for discontinuing or modifying allocated interventions {11b}

Not applicable. This study was determined to not be human subjects research by the Institutional Review Board at the University of New Mexico Health Sciences Center.

### Strategies to improve adherence to interventions {11c}

All peer reviewers, including LIS reviewers, may receive automated, journal-specific reminder emails per the journal’s usual practice. No special instructions or reminders will be sent to LIS reviewers.

### Relevant concomitant care permitted or prohibited during the trial {11d}

Not applicable. This study was determined to not be human subjects research by the Institutional Review Board at the University of New Mexico Health Sciences Center.

### Provisions for post-trial care {30}

Not applicable. This study was determined to not be human subjects research by the Institutional Review Board at the University of New Mexico Health Sciences Center.

### Outcomes {12}

#### Primary outcomes

The primary outcomes will be:

- Quality of reporting as indicated by differences in the proportion of adequately reported searches in first revision manuscripts between intervention and control groups. To be considered adequately reported, four key PRISMA-S [6] reporting items must be adequately reported. Table 1 contains a list of these four key PRISMA-S reporting items with a brief description of each.
- Risk of bias as indicated by differences in the proportions of first revision manuscripts between intervention and control groups with high, low, and unclear bias in ROBIS [38] Domain 2 signaling questions 2.1–2.4. To be considered low risk of bias, all four signaling questions must be answered as Yes or Probably Yes. Table 2 contains a list of these four included ROBIS Domain 2 signaling questions.

**Table 1:** The four PRISMA-S items essential for adequately reporting searches in bibliographic databases, the most common information source used in systematic reviews

PRISMA-S item number	Section	PRISMA-S item	Short explanation
1	Information sources and methods	Name each individual database searched, stating the platform for each.	Is the database and the associated platform for each listed in the manuscript or supplemental materials? Is it clear exactly which database(s) are searched? If multiple databases are searched on one platform, are all of the databases searched listed?
8	Search strategies	Include the search strategies for each database and information source, copied and pasted exactly as run.	Are the full, copied and pasted, unadulterated search strategies from each bibliographic database searched available in the manuscript or supplemental materials? If a generic search strategy is included that is for all databases, is it clear that this search strategy could be reproduced in each database?
9	Search strategies	Specify that no limits were used, or describe any limits or restrictions applied to a search (e.g., date or time period, language, study design) and provide justification for each use.	Does the manuscript text indicate that searches were limited using database limits? Does the manuscript explicitly state that no limits were applied to the search? Does the full search strategy for each database match the text and/or indicate that limits were applied?
13	Search strategies	For each search strategy, provide the date when the last search occurred.	Does the manuscript or supplemental material explicitly state the date that the last search was executed in each bibliographic database?

**Table 2:** ROBIS Domain 2 signaling questions [38]

ROBIS number	Signaling question	Rating
2.1	Did the search include an appropriate range of databases/electronic sources for published and unpublished reports?	Yes/probably yes/probably no/no/no information
2.2	Were methods additional to database searching used to identify relevant reports?	Yes/probably yes/probably no/no/no information
2.3	Were the terms and structure of the search strategy likely to retrieve as many eligible studies as possible?	Yes/probably yes/probably no/no/no information
2.4	Were restrictions based on date, publication format, or language appropriate?	Yes/probably yes/probably no/no/no information

##### Quality of reporting

PRISMA-S was developed as an extension to the main PRISMA reporting guideline to provide more specificity on the details of reporting the search component of a systematic review or related review type [5–8]. Because it was designed to support reporting of complex search methodologies, its checklist items address many aspects of systematic review searches that are not relevant to all systematic reviews. We identified four key items from the PRISMA-S checklist that are essential for reporting searches from bibliographic databases, such as MEDLINE, Embase, and Cochrane CENTRAL, the type of information source used in nearly all of systematic reviews [9, 14].

Assessors will respond “yes” or “no” when evaluating whether each of the four PRISMA-S items is adequately reported. For the quality of reporting primary outcome, all four PRISMA-S reporting items must be adequately reported for the search to be classified as adequately reported. Binary responses were selected because the presence or absence of the four selected PRISMA-S items does not require judgement; the components are either present, or they are not. Binary responses are commonly used in research on search strategy reporting when examining concepts similar to the selected PRISMA-S items [10, 12–15].

##### Risk of bias

ROBIS is a tool designed for assessing the risk of bias in systematic reviews in four domains: study eligibility criteria, identification and selection of studies, data collection and study appraisal, and synthesis and findings [38]. Domain 2, identification and selection of studies, is particularly useful for assessing the potential risk of bias related to the search component of systematic reviews. It includes four signaling questions (see Table 2) specifically related to search comprehensiveness, search methods, search terms/structure, and limits/restrictions that are essential to understanding the risk of bias. The fifth signaling question for ROBIS Domain 2 is related to the process of selection of studies for the review, not the searching component, and is therefore not included in our analysis. We elected to use ROBIS for this study because it is commonly used to assess the risk of bias in systematic reviews, including for search assessment [10], and because it was created through a robust, consensus-based process [38].

Each assessor will assess the four ROBIS signaling questions separately and use them to determine the primary outcome, the overall risk of bias. When evaluating the ROBIS Domain 2 items, the assessors will use the ROBIS instrument’s options. The assessors will respond to each signaling question with “yes,” “probably yes,” “probably no,” “no,” or “no information.” The “no information” category is used only if there is not enough detail to make a judgement. The ROBIS instrument also requests that assessors record notes to support their judgement.

Using these judgements, assessors will then determine the overall bias in this domain as high, low, or unclear. All four questions must have “yes” or “probably yes” answers to receive an overall low risk of bias rating. If any questions are answered as “no information,” the overall rating will be unclear risk of bias. If one or more questions is answered as “probably no” or “no,” the overall rating will be high risk of bias.

#### Secondary outcomes

- Differences in the proportion of papers rejected after the first round of peer review between intervention and control groups
- Differences in the proportion of papers requiring revisions not resubmitted after the first round of peer review between intervention and control groups
- Differences in the proportion of first revision manuscripts for PRISMA-S item 1 between intervention and control groups (see Table 1)
- Differences in the proportion of first revision manuscripts for PRISMA-S item 8 between intervention and control groups (see Table 1)
- Differences in the proportion of first revision manuscripts for PRISMA-S item 9 between intervention and control groups (see Table 1)
- Differences in the proportion of first revision manuscripts for PRISMA-S item 13 between intervention and control groups (see Table 1)
- Risk of bias as indicated by differences in the proportions of first revision manuscripts with high, low, and unclear bias in ROBIS Domain 2 signaling question 2.1 between intervention and control groups (see Table 2)
- Risk of bias as indicated by differences in the proportions of first revision manuscripts with high, low, and unclear bias in ROBIS Domain 2 signaling question 2.2 between intervention and control groups (see Table 2)
- Risk of bias as indicated by differences in the proportions of first revision manuscripts with high, low, and unclear bias in ROBIS Domain 2 signaling question 2.3 between intervention and control groups (see Table 2)
- Risk of bias as indicated by differences in the proportions of first revision manuscripts with high, low, and unclear bias in ROBIS Domain 2 signaling question 2.4 between intervention and control groups (see Table 2)

### Participant timeline {13}

**Fig. 1:**
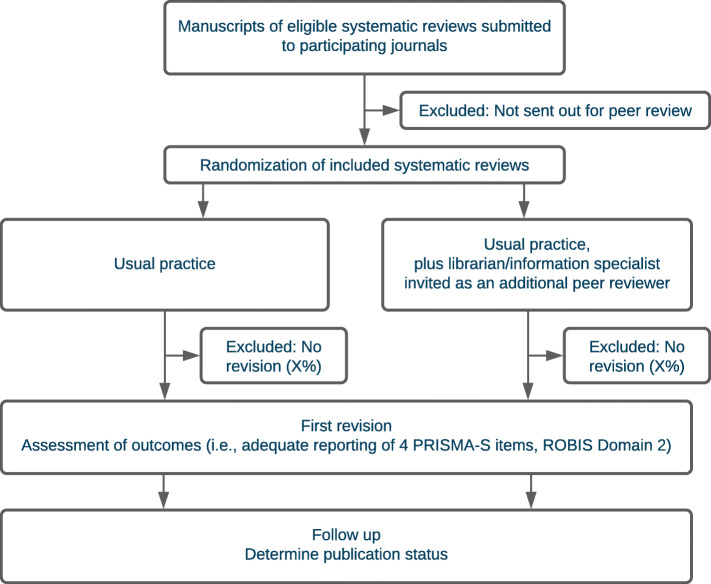
Study process

An overview of the study process is included in Fig. 1.

### Sample size {14}

For a sample size calculation, we hypothesized that approximately 5% of the control group and 20% of the intervention group would adequately report all four key PRISMA-S items in the first revision. This estimation is based on findings from previous studies, though no study so far has looked at these four key PRISMA-S items. Golder et al. found that 4.7% of 277 adverse effects systematic reviews reported the full search strategies and date and language restrictions, but only 0.4% adequately reported the full search strategies, date and language restrictions, and database platform names [39]. Looking at Cochrane Collaboration systematic reviews, Yoshii et al. determined that 0% of studies listed the names of the databases searched, the database platforms, the date the search was run, the years covered by the search, the full search strategy, and any language restrictions [40]. More recently, Koffel and Rethlefsen found that 0% of 272 pediatrics, cardiology, and surgery systematic reviews adequately reported the database platform, specific year for first and last years searched, the date the search was conducted and updated, provided a full search strategy, and indicated if limits were used [15]. The same study found that 14% did report the database searched, first and last years searched, the complete search strategies, and whether limits applied to all databases searched, but also found that only 6% named the database platform [15].

To assess whether the journals in this study would similarly have baseline low quality of search reporting, the 10 most recently published systematic reviews, as of August 29, 2020, in four of the BMJ Publishing Group’s journals, *BMJ*, *BMJ Open*, *British Journal of Sports Medicine*, and *Heart*, were examined for adequate reporting for the four key PRISMA-S items. Of the 40 articles examined, only 2, or 5%, adequately reported all four items. This was in line with findings from previous studies.

To demonstrate a significant between groups difference of 15 percentage points with a power of 80% and alpha at 0.05, a total of 150 first revision manuscripts will be required, 75 in each arm. The final sample will be based on the number of first revision manuscripts; therefore, we will keep randomizing submissions until 75 first revision manuscripts in each arm have been received by the journals.

### Recruitment {15}

To identify eligible manuscripts, MLR will screen automated submissions lists from ScholarOne at least twice a week for manuscripts meeting criteria. The automated Excel reports will contain manuscript title, date of original submission, abstract, number of reviewers invited, first decision and date, and latest decision and date. Potentially eligible manuscripts will first be identified using searches for the following keywords and their variants in the title and abstract variables:

Systematic review, Scoping review, Realist review, Mixed methods review, Meta-analysis, Rapid review, Umbrella review, Review of reviews, Systematic map, Mapping review, Evidence synthesis, Overview

If eligibility is not clear from the title and abstract, the full text will be reviewed. We will then screen for these potentially eligible manuscripts in another ScholarOne report listing all manuscripts sent out for review (i.e., the variable “number of invited reviewers” will be greater than zero). At that point, MLR will randomize the manuscript to the intervention or control arm based on manuscript ID. Each manuscript in the intervention arm will be color flagged and labeled in the ScholarOne system for easy identification by editors, researchers, and administrators.

Manuscript recruitment will complete when the desired sample size has been achieved.

## Assignment of interventions: allocation

### Sequence generation {16a}

Manuscripts meeting the eligibility requirements will be randomized to the intervention or control group (1:1) soon after the first peer reviewer is invited to review. This will be indicated in ScholarOne when the number of “peer reviewers invited” variable is greater than zero. Manuscripts will be stratified by journal and randomized using permuted block randomization in blocks of four. The Study Randomizer app will be used to generate the sequence [41].

If multiple eligible manuscripts are identified on the same day, the manuscripts will be randomized in the sequence of their time of submission.

### Concealment mechanism {16b}

Each time a new manuscript meets eligibility criteria, MLR will go to the Study Randomizer dashboard to enroll a new manuscript by selecting the journal for stratification and entering the manuscript ID (supplied by the ScholarOne system). The randomization for that manuscript will be provided by the Study Randomizer tool [41].

### Implementation {16c}

MLR will generate the allocation sequence using Study Randomizer [41], determine eligibility, and assign manuscripts to interventions.

## Assignment of interventions: blinding

### Who will be blinded {17a}

Manuscript authors and co-reviewers will be masked to the intervention. Editors will not be blinded to the identity of the peer reviewers submitting reports and will be aware that a manuscript is part of the study.

All manuscripts will be assigned a unique study identification number for masking. Outcome assessors will be blinded to the group allocation and will not have access to the ScholarOne system.

### Procedure for unblinding if needed {17b}

Not applicable. Unblinding will not be required.

## Data collection and management

### Plans for assessment and collection of outcomes {18a}

The outcomes will be assessed independently by two blinded outcome assessors per revised manuscript. The outcome assessors have expertise in reporting the search component of systematic reviews as well as methodological expertise designing and executing searches for systematic reviews. Disagreements in assessments will be resolved through consensus or, if necessary, by inviting the third reviewer to help resolve disagreement. We will provide assessors with PRISMA-S guidance and ROBIS documentation prior to piloting data extraction forms using 5–10 sample systematic reviews from outside the study population. Any inconsistencies in interpretation will be discussed and the data extraction forms modified for clarity and usability. All variables that will be collected are in LibPeerRevDataCollectionTable2021-06-17.docx: https://osf.io/78ujt/

The editorial decision (reject/revise) after the first round of peer review and final editorial decision (accept/reject) will be gathered by MLR using the ScholarOne systems and will not be blinded.

### Plans to promote participant retention and complete follow-up {18b}

Manuscripts will be randomized when submitted for peer review, but only manuscripts that have a first revision will be analysed. We will track manuscript rejection after the first peer review.

### Data management {19}

Data from the journals’ editorial systems will be extracted (by MLR) and maintained on a secure, password-protected Google Drive folder hosted at the BMJ Publishing Group. The folder will be accessible only to MLR and SS; Google Drive complies with all General Data Protection Regulation (GDPR) requirements. Assessors will access the anonymized study manuscripts through a separate secure, password-protected Google Drive folder hosted at the BMJ Publishing Group. Data collected from the assessors will be collected and stored using a Google Drive folder hosted at the BMJ Publishing Group to facilitate resolution of outcome assessment conflicts between assessors.

Derived/aggregated anonymized data will be shared with the research community upon completion of the research using a publicly accessible data repository.

### Confidentiality {27}

MLR and MZ will be given access to identifiable data from ScholarOne editorial systems for participating journals under a confidentiality agreement. SS is a full-time employee of *BMJ* and regularly undertakes research into the publication process. All outcome assessors will be subject to a confidentiality agreement before assessing blinded manuscripts. Access to personally identifiable information about librarian/information specialists in the Librarian Peer Reviewer Database is made accessible to any journal editor without restriction in order to facilitate the peer review process and is not subject to a confidentiality agreement.

### Plans for collection, laboratory evaluation, and storage of biological specimens for genetic or molecular analysis in this trial/future use {33}

Not applicable. This study does not collect biological specimens.

## Statistical methods

### Statistical methods for primary and secondary outcomes {20a}

We will begin by assessing differences between first revision manuscripts in the control and intervention arms by examining the proportions of adequately reported searches (all four PRISMA-S items reported adequately) in each group using chi-square tests or Fisher’s exact tests. We will also examine the proportions of adequate reporting of each of the four PRISMA-S items (Table 1) to determine whether specific items may be reported adequately more frequently in one of the groups using chi-square tests or Fisher’s exact tests. We will calculate the effect sizes using Cramer’s V [42].

We will use this same method to assess differences in the risk of bias demonstrated in the first revision manuscripts. The risk of bias assessment will be a global ROBIS Domain 2 rating (high, low, or unclear risk). We will also use chi-square tests or Fisher’s exact tests to examine whether there are differences between intervention and control groups for each of the four signaling questions. We will calculate the effect sizes using Cramer’s V.

Bivariate and multivariate logistic regression will be used to investigate potential associations between the global and individual PRISMA-S and ROBIS Domain 2 ratings and potential confounders. These include number of authors, journal the manuscript was submitted to, librarian/information specialist involvement in the systematic review, librarian/information specialist authorship of the systematic review, citation of the main PRISMA Statement or one of its extensions, the citation of an alternate reporting guideline or conduct guideline (e.g., the Cochrane Handbook, MOOSE, etc.), or the citation of the PRESS Guideline. We will first conduct a series of bivariate logistic regressions for each predictor and then conduct a multivariate logistic regression by including all predictors in a single model. This will allow us to test the independent association of each variable as well as control for the others. We will report odds ratios with 95% confidence intervals for these analyses. We will conduct primary analyses using an intention-to-treat-model.

We will also examine differences in the proportion of papers rejected after the first round of peer review. This analysis will be conducted using chi-square or Fisher’s exact tests. We will calculate the effect size using Cramer’s V.

We will also assess the level of initial agreement between assessors using Cohen’s kappa [43].

### Interim analyses {21b}

Not applicable. We do not plan to conduct interim analyses.

### Methods for additional analyses (e.g., subgroup analyses) {20b}

We will conduct an as-treated sensitivity analysis if one or more manuscripts in the intervention group receive no review from invited LIS reviewers. In addition, it is possible that manuscripts could be sent to librarians or information specialists who are not flagged for the study, particularly in the control arm. If, upon examination, there are any librarian/information specialist peer reviewers invited who are not invited as part of the intervention, we will conduct sensitivity analyses.

### Methods in analysis to handle protocol non-adherence and any statistical methods to handle missing data {20c}

We will conduct primary analyses using an intention-to-treat-model. Missing data from outcome assessment will be returned to outcome assessors for completion.

### Plans to give access to the full protocol, participant-level data, and statistical code {31c}

Aggregate and/or anonymized data will be shared using the Open Science Framework. Peer review reports and manuscripts are subject to confidentiality agreements and will not be shared. Public accessibility of manuscripts and peer review reports is based on publication of a manuscript and journal editorial policy. Accepted publications in *BMJ* and *BMJ Open* provide pre-publication histories, including open peer review reports, manuscript versions, and editorial comments.

## Oversight and monitoring

### Composition of the coordinating center and trial steering committee {5d}

Not applicable. We do not have a coordinating center or a trial steering committee.

### Composition of the data monitoring committee, its role, and reporting structure {21a}

Not applicable. We do not have a data monitoring committee.

### Adverse event reporting and harms {22}

Not applicable. This study was determined to not be human subjects research by the Institutional Review Board at the University of New Mexico Health Sciences Center.

### Frequency and plans for auditing trial conduct {23}

Not applicable. No auditing or data monitoring is planned.

### Plans for communicating important protocol amendments to relevant parties (e.g., trial participants, ethical committees) {25}

The protocol will be updated as needed and amendments shared on the Open Science Framework project page for this study.

## Dissemination plans {31a}

The results of the study will be published as preprints and will be submitted for publication in open access peer-reviewed journals regardless of study outcome(s).

## Discussion

We sought patient and public involvement to review the protocol, with special emphasis on assessing whether the outcomes are meaningful for patients and the public. We recruited individuals from amongst those who volunteer as patient and public peer reviewers for the BMJ Publishing Group. One individual agreed to participate. When the study is underway, the patient and public representative volunteer will be invited to participate in study team meetings to provide additional feedback during the conduct of the study.

## Trial status

This is protocol version 1. Recruitment has not yet begun. We anticipate that it will begin in late 2021.

## Abbreviations

LIS: Librarian or information specialist
MOOSE: Meta-analysis Of Observational Studies in Epidemiology reporting guideline
PRESS: Peer Review of Electronic Search Strategies instrument
PRISMA: Preferred Reporting Items for Systematic reviews and Meta-Analyses reporting guideline
PRISMA-S: Preferred Reporting Items for Systematic reviews and Meta-Analyses literature search extension
ROBIS: Risk of Bias in Systematic reviews instrument
